# Characterization of a broad-spectrum antifungal strain, *Streptomyces graminearus* STR-1, against *Magnaporthe oryzae*

**DOI:** 10.3389/fmicb.2024.1298781

**Published:** 2024-04-08

**Authors:** Wenyuan Shen, Renju Liu, Jiazheng Wang, Maolan Yang, Tuo Qi, Guosong Shu, Min He, Xuewei Chen

**Affiliations:** State Key Laboratory of Crop Gene Exploration and Utilization in Southwest China, Sichuan Agricultural University at Wenjiang, Chengdu, Sichuan, China

**Keywords:** biocontrol, *Magnaporthe oryzae*, antifungal, fungal pathogens, *Streptomyces*

## Abstract

Fungal diseases such as the devastating rice blast pose severe threats to crop production worldwide. Biological control of crop diseases caused by fungal pathogens is an environment-friendly approach for safeguarding crop production. But the insufficient availability of microbial agents effective against various fungal diseases has hampered the development of green production in crops. In this study, we identified a broad-spectrum antifungal bacterium, *Streptomyces graminearus* STR-1, showing antagonistic activity to diverse fungal pathogens including *Magnaporthe oryzae*, *Rhizoctonia solani*, *Fusarium graminearum*, *Ustilaginoidea virens*, and *Bipolaris maydis*. Its antifungal activity was relatively stable and less affected by temperature and pH. Evaluation of the biocontrol activity of STR-1 revealed that STR-1 prevented and controlled rice blast disease via eliciting plant immunity and suppressing fungal infection-structure development. STR-1 broth extract inhibited spore germination, likely through inhibiting protein synthesis. Combining LC–MS and chromatography analysis of the antimicrobial compounds purified from STR-1 broth extract, together with decoding STR-1 genomic sequence, we identified 4-oxo-4-[(1-phenylethyl)amino]but-2-enoic acid, 1,3,5-Trimethylpyrazole and SMA-1 as the potential main STR-1 secondary metabolites associated with its antifungal effects. This study suggests that bacterial strain STR-1 could be used for identifying highly effective and broad-spectrum secondary metabolites for containing rice blast and other crop diseases. The application of the active compounds offers a promising measure to tackle fungal disease.

## 1 Introduction

Global agricultural production and crop quality are heavily affected by fungal diseases (Almeida et al., 2019; Fisher and Garner, 2020). Rice (*Oryza sativa*) is one of the most important food crops, feeding half of the world’s population, but its production is severely threatened by rice blast disease (Liu et al., 2021). *Magnaporthe oryzae*, as the causal agent of blast disease, is an invasive fungus that can infect different parts of rice plants to cause leaf blast, stem blast, panicle blast, and grain blast (Neftaly et al., 2021). Rice blast disease leads to leaf withering, wilting and failure in spikelet development, and eventually rice yield reduction or even death (Li et al., 2019).

Biological control of fungal diseases is attracting intensive attention in research and application, because it is one of most sustainable and environmentally friendly disease control measures (Martínez-Hidalgo et al., 2019). Biological control agents of microbes or plants can produce various metabolites as bioactive fungicides to inhibit the growth and infection of pathogens (Maťátková et al., 2022; Ayilara et al., 2023). These bioactive fungicides prevent the occurrence of fungal diseases through multiple mechanisms, including inhibiting the growth, reproduction, and infection of pathogenic microbes, altering host plant resistance, and activating the plant’s defense responses (Wei et al., 2021; Ahmed et al., 2022; Liu et al., 2022). Several microbes including fungi and bacteria have been reported to harbor biological control capability by inhibiting the growth of the blast fungus *M. oryzae*. For instance, *Bacillus*, *Streptomyces*, and *Pseudomonas* are effective in controlling blast diseases (Park et al., 2006). To protect crop production from severe diseases, it is an urgent need to develop effective and broad-spectrum biological control agents.

*Streptomyces graminearus* is a gram-positive bacterium belonging to the genus *Streptomyces*. It is widely found in soil, usually in the form of branched mycelium (Komaki, 2023). *S. graminearus* can produce diverse antibiotics displaying a broad spectrum of antimicrobial activity. It has therefore been used to fight plant diseases caused by various pathogenic bacteria, fungi, and other microorganisms (Rahila et al., 2023). Previous studies have shown that different *Streptomyces* species can inhibit mycelial growth of fungal pathogens ranging from 22 to 98%, such as *S. palmae* PC 12, *S. vinaceusdrappus*, *S. philanthi* RM-1-138, *S. globisporus* JK-1, *S. griseofuscus*, *S. hygroscopicus*, *S. griseus* strain SKB2.14, *S. hygroscopicus* strain OsiSh-2, and *Streptomyces* sp. (Chakraborty et al., 2021). *Streptomyces* species can also produce several secondary metabolites with strong inhibitory effects on pathogenic microbes. For instance, staurosporine synthesized by *S. roseoflavus* strain LS-A24 suppressed mycelial growth of various fungal pathogens with minimum inhibitory concentration of 1-50 mg/mL (Park et al., 2006). Despite the isolation of various *Streptomyces* strains with outstanding potential in biological control, the identification of bioactive metabolites from these strains is relatively insufficient and has thus slowed the development of new fungicides.

In this study, we isolated a new *S. graminearus* strain STR-1 that exhibited antifungal properties against various phytopathogenic fungi including *M. oryzae*. Combining a metabolomic and genomic approach, we characterized the metabolite composition of STR-1 to discover that secondary metabolites produced by STR-1 were able to suppress spore germination and hyphal growth of *M. oryzaea*. These findings hold significant promise for sustainable agriculture and the development of new fungicides.

## 2 Materials and methods

### 2.1 Isolation and identification of biocontrol bacterium

The biocontrol bacterium was obtained from rice rhizosphere soil by using a co-culture method against *M. oryzae* in CM medium as previously described. The rice rhizosphere soil was collected from a rice growing area, Wenjiang District, Chengdu in Sichuan Province of China. The bacterial strain STR-1, which shows a notable inhibitory effect on growth of *M. oryzae*, was used for further analysis. The DNA fragment encoding 16S RNA was amplified by the specific primer (27F: AGAGTTTGATCMTGGCTCAG; 1492R: CGGTTACCTGTTACGACTT) and the phylogeny were performed by the neighbor-joining (NJ) with MEGA 7.0 software (RRID: SCR_011920)^1^ (Kumar et al., 2016).

### 2.2 Genome sequencing, assembly, and annotation of STR-1

The genomic sequence assembly was obtained with the Whole Genome Shotgun (WGS) strategy. First, a DNA Paired-end (PE) library of about 400 bp was constructed with Illumina TruSeq DNA Sample Prep kit (Cat. 92122) by following manufacture’s protocol, and sequenced on the Illumina NovaSeq sequencing platform (Bankevich et al., 2012). The adapter sequence and low-quality reads were removed by FastQC and Adapter Removal. Then genome assembly was performed by A5-Miseq and SPAdes and corrected by Pilon. To annotate genome assembly, GeneMarkS (Besemer et al., 2001) software was used to predict protein-coding genes with an HMM model. Barrnap and tRNAscan-SE were used to predict rRNA and tRNA, respectively. Diamond software was used to complete the sequence alignment of protein-coding genes against NCBI NR database, Swiss-Port database, and COG databases (*E*-value <1.0e-6) (Buchfink et al., 2015). KEGG (Kyoto Encyclopedia of Genes Genomes) terms were annotated by KASS based on prokaryotes type and bi-directional best hit rule. GO (Gene Ontology) terms were identified by BLAST2GO by default parameters.

### 2.3 Identification of secondary metabolites biosynthesis genes

Gene cluster analysis of secondary metabolite biosynthesis was carried out by the online antiSMASH 6.0 software.^2^ Analysis parameters were set to include whole-genome analysis, with “KnownClusterBlast” and “SubClusterBlast” options selected for comparative analysis against known gene clusters. Then novel clusters were defined by comparing with known clusters in the database. For each predicted gene cluster, antiSMASH provided further prediction of functional genes and putative secondary metabolites (Blin et al., 2021).

### 2.4 Bacterial strains and growth conditions

Plant pathogenic fungi were collected and stored following standard methods, including *Ustilaginoidea virens*, *Fusarium graminearum*, *Magnaporthe oryzae*, *Rhizoctonia solani*, and *Bipolaris maydis*. The blast fungus *M. oryzae* strain Guy11 were used throughout this study. Guy11 was grown on CM medium or tomato oat medium at 28°C for 10 days and its spore suspension was prepared for inoculating rice plants as previously described (Yang C. et al., 2023).

### 2.5 Crude extraction of STR-1 fermentation broth

The extraction of STR-1 fermentation broth products was conducted following a previous report. Under the condition of reduced pressure at 45°C, culture filtrates of STR-1 grown in PDA liquid medium were concentrated to a dark-brown tarry residue, which was dissolved by distilled H_2_O to a concentration of 50 g/L. Thereafter, the dark-brown tarry residue dissolved in water was supplemented with equal volume of solvent ethyl acetate, petroleum ether, or methylene chloride and mixed thoroughly. The separated organic phase was processed with a rotating vacuum evaporator at 45°C for producing paste extracts, which were subsequently dissolved with distilled H_2_O to a concentration of 1 mg/mL for testing antifungal activity. The extracts of ethyl acetate displayed an inhibitory effect on fungal growth. Thus, the paste extracts of ethyl acetate were dissolved with methanol at a concentration of 1 mg/mL for the next round of extraction by silica gel column chromatography. Using thin layer chromatography (TLC), the optimal condition of developing solvent for separation was determined as a combination of n-hexane and ethyl acetate at a ratio of 15:1 (Yang C. et al., 2023).

### 2.6 Column chromatography assay

For the column chromatography experiments, silica gels with the size of 200 mesh were added into the column through the funnel. For ensuring compact loading, silica gels were added continuously and uniformly to a final volume of 250 mL. When the height of silica gels reached about three-quarters of the column, the lower piston of the air pump was opened to compress the column bed volume. A layer of quartz sand at a thickness of 1 ~ 2 mm was added onto the surface of the silica gel column. The STR-1 paste extracts of ethyl acetate dissolved with methanol were poured into the silica gel column under the vacuum condition by pressurizing with an air pump to allow the extract solution stream into silica gel for about 2 ~ 3 cm thickness. Subsequently, developing solvent (n-hexane: ethyl acetate = 15:1) was added into the column at the flow rate of 1 ~ 2 drops/s, and a total of 500–750 mL of developing solvent was used for separating the extracts. In the process of separation, 20 mL test tubes were used to collect samples eluted from the column. One tube of eluted sample was further detected by TLC to determine which of the samples correspond to the five fragments F1, F2, F3, F4, or F5. Through silica gel column chromatograph, the extracts of ethyl acetate were separated into F1, F2, F3, F4, and F5, and each of the extracted fractions was applied in the following fungal antagonism assay for identifying separated fractions with the most potent antifungal property.

### 2.7 Test of antagonistic effect of STR-1 crude extract on fungal growth

For fungal antagonism assays, STR-1 strain was cultured in PDA liquid medium at 200 rpm in a 28°C constant temperature shaker for 5 days. To prepare the STR-1 bacterial fermentation broth, STR-1 grown in PDA liquid medium was centrifuged at 4°C at 11,000 r/min for 20 min to collect the supernatant that contains fermented products of STR-1. The supernatant was then filtered and sterilized with 0.22 μm filter to obtain filtrate fermentation solution. Next, CM medium plate used for growing fungal strain was supplemented with filtrate fermentation solution by indicated dilution factors (10×). The PDA liquid medium without inoculation of STR-1 was similarly filtered and applied into CM medium plate as a control. A 7 mm diameter agar block grown with *M. oryzae* or other fungal strains was inoculated onto the center of CM plates containing filtrate fermentation solution of STR-1. The inoculated plates were cultured for 7 days in a 28°C incubator as previously described (Yang C. et al., 2023). The growth of *M. oryzae* was monitored and recorded throughout the growth period. The inhibition of fungal growth was quantitated by the following formula: inhibitor rate = [(diameter of *M. oryzae* colony in the control-diameter of *M. oryzae* colony in the treatment) ÷ (diameter of *M. oryzae* colony in the control)] × 100%.

### 2.8 Examination of pathogenicity of *Magnaporthe oryzae*

The inoculation of rice leaves with *M. oryzae* was conducted as previously described by punch inoculation or spraying inoculation (Li et al., 2017). In punch inoculation assay, rice leaves of a susceptible cultivar Lijiangxintuanheigu (LTH) were cut from seedlings at the three-leaf stage and put on the surface of 0.1% 6-Benzylaminopurine (6-BA, pH = 7.0) solution. Fungal conidia were collected by flooding Guy11 strain grown on CM medium with sterile water and were filtered through miracloth to remove mycelia and impurities. Conidial suspension of Guy11 was adjusted to a concentration of 1 × 10^5^ /mL. Then five microliter of conidial suspension was used for punch inoculation of detached rice leaves with wounding the site, which was achieved by pressing the leaves with tip of a 10 μL transferpettor. In spraying inoculation, rice seedlings at the three-leaf stage were inoculated with conidial suspension and kept in an artificial chamber under darkness for 12 h before transferring into the 12 h light/12 h dark environment. After 5–7 days of incubation, the blast lesions developed on rice leaves were recorded for each treatment. Lesion length was counted for the punch inoculation assay, while the lesion number was counted for the spraying inoculation assay. Photographed lesion length and number was measured by ImageJ (RRID:SCR_003070).^3^

### 2.9 Determination of STR-1 extracts on infection-related development of *Magnaporthe oryzae*

The effects of STR-1 fermentation broth on infection-related development of *M. oryzae* were examined by microscopic observation of conidial germination, appressorium formation, and maturation using Guy11 conidial suspension as previously described (He et al., 2018). Conidial suspension prepared as described was supplemented with the control solvent methanol. Each separated extracts of F1, F2, F3, F4, and F5 was individually added into the conidial suspension to a proper concentration, and the solvent methanol was added as a control. The conidial suspension supplemented with extracts was inoculated onto a hydrophobic coverslip (fisherbrand microscope cover glass Cat. No. 12540C), incubated in a moisturizing petri dish at 28°C. The germination of conidia and the formation of appressorium were observed under a microscope (Zeiss AXIO Imager. M2/ApoTome 3525004037) at 0, 4, 12, 24, and 36 h post inoculation (hpi).

### 2.10 Untargeted metabolomics analysis

For identifying specialized metabolites produced by STR-1, the extracts of F5 separated by silica gel column were analyzed by ultra-performance liquid chromatography tandem-mass spectrometry (UPLC–MS/MS) analysis at Metware Biotechnology Co., Ltd., China. In brief, an aliquot of 100 mg of extract sample was dissolved in 1 mL of 70% methanol, 3 mm steel balls were added, and this was processed with an automatic sample rapid grinder (jxfstprp-48, 70 Hz) for 3 min, and then cooled with low-temperature ultrasound (40 KHz) for 10 min. After centrifugation at 12000 rmp for 10 min at 4°C, the supernatant was collected and diluted by 2–100 times with methanol followed by the addition of 10 μL of 100 μg/mL internal standard. The processed sample was passed through a 0.22 μm PTFE filter for on-board detection on Thermo Vanquish UHPLC coupled with Q-Exactive HF (Thermo Fisher Scientific) with chromatographic column Zorbax Eclipse C18 (1.8 μm × 2.1 mm × 100 mm, Agilent technologies). The chromatographic separation conditions were as follows: the column temperature is 30°C; the flow rate is 0.3 mL/min; 0.1% fomic acid solution was used as mobile phase composition A, and pure acetonitrile as mobile phase composition B; injection volume was 2 μL; and the auto sampler temperature was 4°C.

### 2.11 RT-qPCR assay

The three-leaf stage seedlings of rice susceptible cultivar LTH were sprayed with 10 mL of separated extract of F5. The seedlings sprayed with water were used as a control. Following spraying inoculation, rice leaves were collected at 0 h, 12 h, and 24 h for RT-qPCR analysis. Rice total RNA was extracted from leaves using Trizol agent after grounding into powder by liquid nitrogen. Reverse transcription of RNA was performed using TaKaRa’s reverse transcription kit (Yang C. et al., 2023), and quantitative PCR was then conducted with specific primer pairs for detecting the expression levels of pathogenesis-related genes (PRs) and other defense-related genes (Table 1). Relative gene expression was normalized by comparing with the expression of *Ubiquitin* and analyzed using the 2^−ΔΔCT^ method (Park et al., 2012). The data was indicated as mean ± s.d.

**Table 1:** The nucleotide sequence for the primers used for PRs and other defense-related genes.

Primer	Sequence (5’ to 3’)
UBQ5F	AACCAGCTGAGGCCCAAGA
UBQ5R	ACGATTGATTTAACCAGTCCATGA
OsPR1a-F	CGTCTTCATCACCTGCAACT
OsPR1a-R	TGTCCATACATGCATAAACACG
OsPR10-F	CTCATCCTCGACGGCTACTT
OsPR10-R	ATCAGGAAGCAGCAATACGG
OsKS4-F	TCGCATTGCGTGTGCAA
OsKS4-R	TTGGAACTTCCGACATCGAAA

### 2.12 Statistical analysis

For analyzing the statistical significance of difference for our experiments, the *p* value was calculated using the two-sided Student’s *t*-test and Microsoft Excel v.15.0. All values are presented as mean ± s.d. and the number of samples is indicated in the legend. All experiments were repeated at least twice to confirm reproducibility.

## 3 Results

### 3.1 Identification of a biocontrol strain STR-1

**Figure 1:**
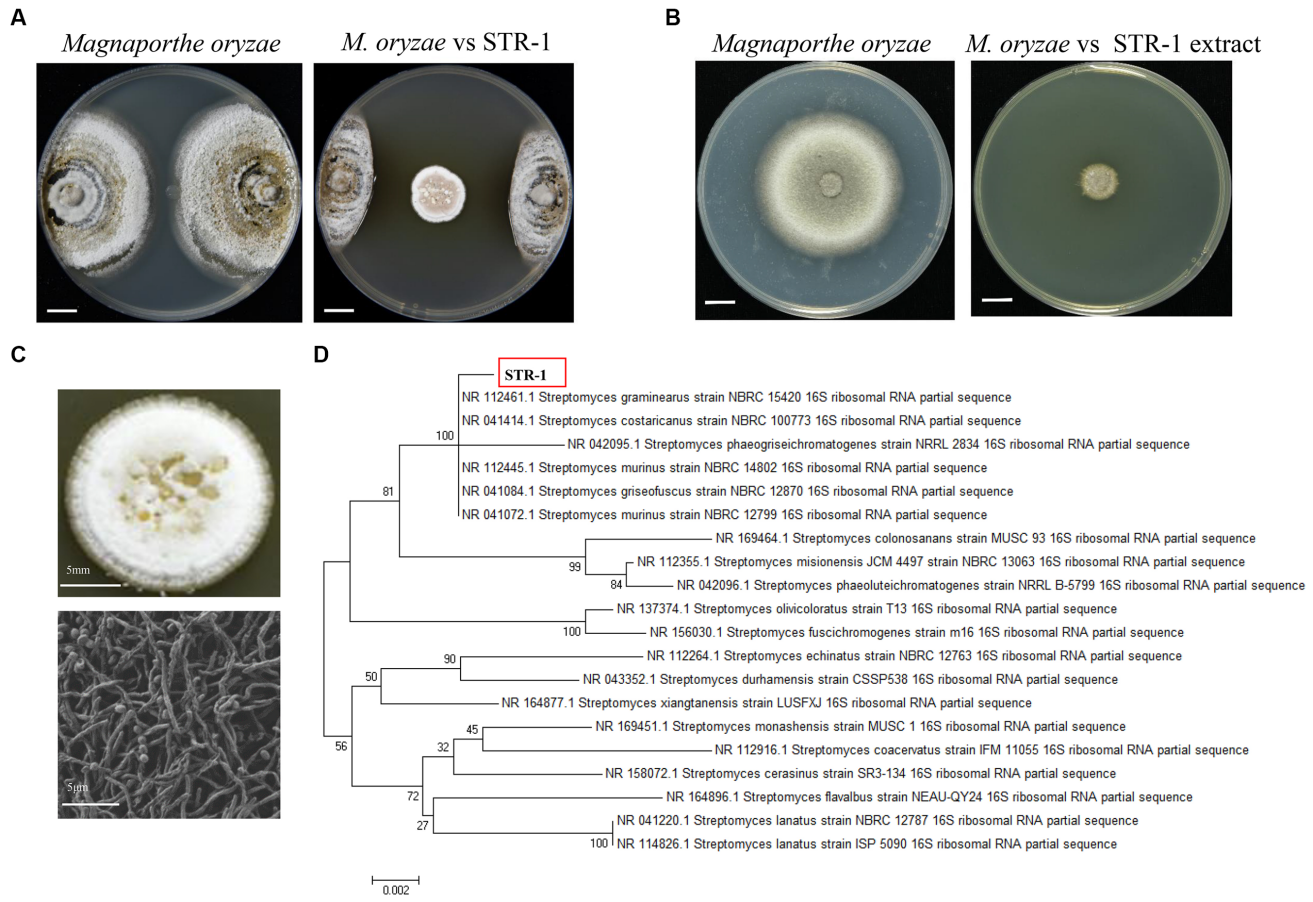
Identification of strain STR-1. **(A)** Antagonistic inoculation of STR-1 was applied to *S. graminearus* colonies grown on plates containing the solid medium PDA, and incubated at 28°C for 6 days, following which the colonies were photographed. **(B)** Effects of extract isolated with 10% of ethanol on mycelial growth of *M. oryzae*. **(C)** Colony morphology and scanning electron microscopy observation of strain STR-1. **(D)** Phylogenetic tree of strain STR-1 based on the 16S rRNA sequence. The tree was constructed using the neighbor-joining method in the MEGA software. The level of bootstrap support (1,000 repetitions) was indicated at all nodes.

A bacterium with antifungal activity was discovered in the laboratory on the rice blast fungus culture medium supplemented with filtrates of rice rhizosphere soil. Crossing-culture assay against *M. oryzae* strain Guy11 displayed a clear inhibition zone as shown in Figure 1A. The fermentation broth of the biocontrol bacteria also displayed a strong antagonistic activity. The growth diameter of Guy11 was 49.1 ± 1.1 mm in CM agar medium, whereas its diameter was reduced to 12.3 ± 0.5 mm when CM agar medium was supplemented with 10% fermentation broth of this strain (Figure 1B).

We further examined the nature of the potential antifungal substance in the fermentation broth of this bacterial strain. The fermentation broths were treated with temperatures as high as 130°C and then used in crossing-culture assay. The fermentation broth treated with high temperature still showed strong inhibitory effects on the growth *M. oryzae* (Supplementary Figure S1A), indicating the tolerance of active anti-fungal substance to high temperature. When the fermentation broth was supplemented into CM agar medium under variable pH values (extreme acid or basic pH), the growth of *M. orzyae* was similarly inhibited by fermentation broth at different pH (Supplementary Figure S1B), indicating the tolerance of active anti-fungal substance to extreme pH.

The morphological properties and molecular biological identification based on 16S rRNA were further analyzed; this biocontrol bacterium forms a round and white colony on CM, while the back will form a red wheel. Electron microscope scanning showed that the mycelium was filamentous with oval spores (Figure 1C). Phylogenetic analysis demonstrated that this bacterium was *S. graminearus*, which shares a close relationship with strain NBRC 15420. This strain was therefore named STR-1 (Figure 1D). Multilocus sequence analysis (MLSA) is a powerful method for species identification as well as the elucidation of phylogenetic relationships in the genus *Streptomyces*. Multigene phylogenetic analysis with gene sequences of *AtpD*, *GyrB*, *RecA*, *RpoB*, and *TrpB* from *Streptomyces* species confirmed that this STR-1 belongs to *Streptomyces graminearus* (Supplementary Figure S2).

### 3.2 Broad antifungal activity of STR-1

**Figure 2:**
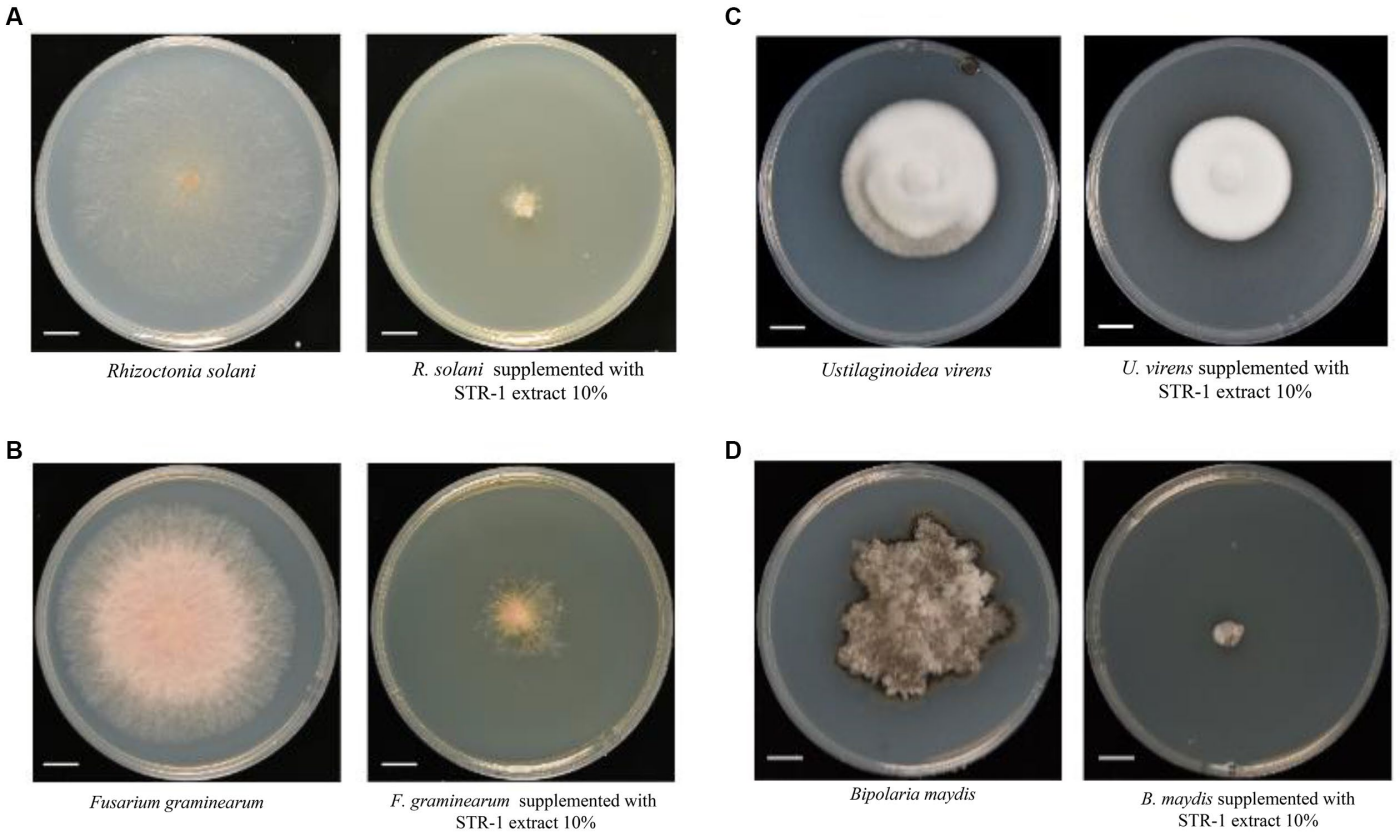
Antifungal activity of strain STR-1 and its extract against the phytopathogenic fungi. Fermentation products of STR-1 grown in liquid PDB medium were supplemented into the PDA plate and used for determining their inhibitory effects on growth of different fungal pathogens including *Rhizoctonia solani* **(A)**, *Fusarium graminearum* **(B)**, *Ustilaginoidea virens* **(C)**, and *Bipolaris maydis* **(D)**, Bar = 1 cm.

We further tested whether STR-1 harbored broad-spectrum antifungal activity. As shown in Figure 2, the plate colony diameter of *Rhizoctonia solani*, the causal agent of rice sheath blight disease, was 73.7 ± 2.1 mm in PDA medium, but was notably reduced into 10.8 ± 0.9 mm in PDA containing 10% PDB fermentation broth of the biocontrol bacterium STR-1. We also measured the effects of STR-1 on the growth of fungal pathogen *Fusarium graminearum*, which causes severe head blight disease in wheat. When quantitating the fungal growth on PDA, the plate colony diameter of *Fusarium graminearum* was 73.3 ± 1.7 mm, but was decreased into 22.1 ± 1.7 mm in the presence of 10% PDB fermentation broth of STR-1. With respect to the fungal pathogen *Ustilaginoidea virens* causing rice false smut disease, we obtained similar results to those shown in Figure 2. The plate colony diameter of *U. virens* was 46.2 ± 2.1 mm in PDA medium but was reduced into 36.4 ± 1.2 mm by exposure to 10% PDB fermentation broth of STR-1. Moreover, fermentation broth of STR-1 displayed strong inhibitor effect on the growth of the *Bipolaris maydis*, the southern corn leaf blight disease agent. *B. maydis* growth was 52.3 ± 10.5 mm in PDA medium but diminished into 8.1 ± 4.3 mm in the presence of 10% PDB fermentation broth of STR-1. Hence, the bacterium STR-1 identified in our study can be used as a broad-spectrum antifungal biocontrol agent and is valuable for further study.

### 3.3 Draft genome sequencing analysis of STR-1

The draft genome of strain STR-1 was sequenced and 9,904,202 high-quality reads including 1,453,805,695 bp with 71.79% G + C content were obtained. This genome consisted of seven rRNA genes, 68 tRNA, and 7,399 ORFs (Supplementary Tables S1–S5). Prediction of protein-coding gene from its genome showed that 7,224 genes were annotated while 2,138 genes were clustered in function unknown COG categories (Supplementary Figure S3A). GO and KEEG classification is shown in Supplementary Figures S3B,C and Supplementary Table S6.

As far as we know, there were no genome sequence of *S. graminearus* reported previously in NCBI database and EZbiocloud database (Yoon et al., 2017). The genome sequences of STR-1 have been deposited in NCBI under the BioProject ID PRJNA1047497. Furthermore, analysis of its genome with antiSMASH 6.0 online software (Blin et al., 2021) showed STR-1 harbors gene clusters that can produce diverse antifungal secondary metabolites, such as 4-Aminobutyric acid, 3-(tert-butyl)-1-methyl-4,5-dihydro-1H-pyrazol-5-one, and SMA-1 (Supplementary Table S7).

### 3.4 Control of rice blast disease by STR-1

**Figure 3:**
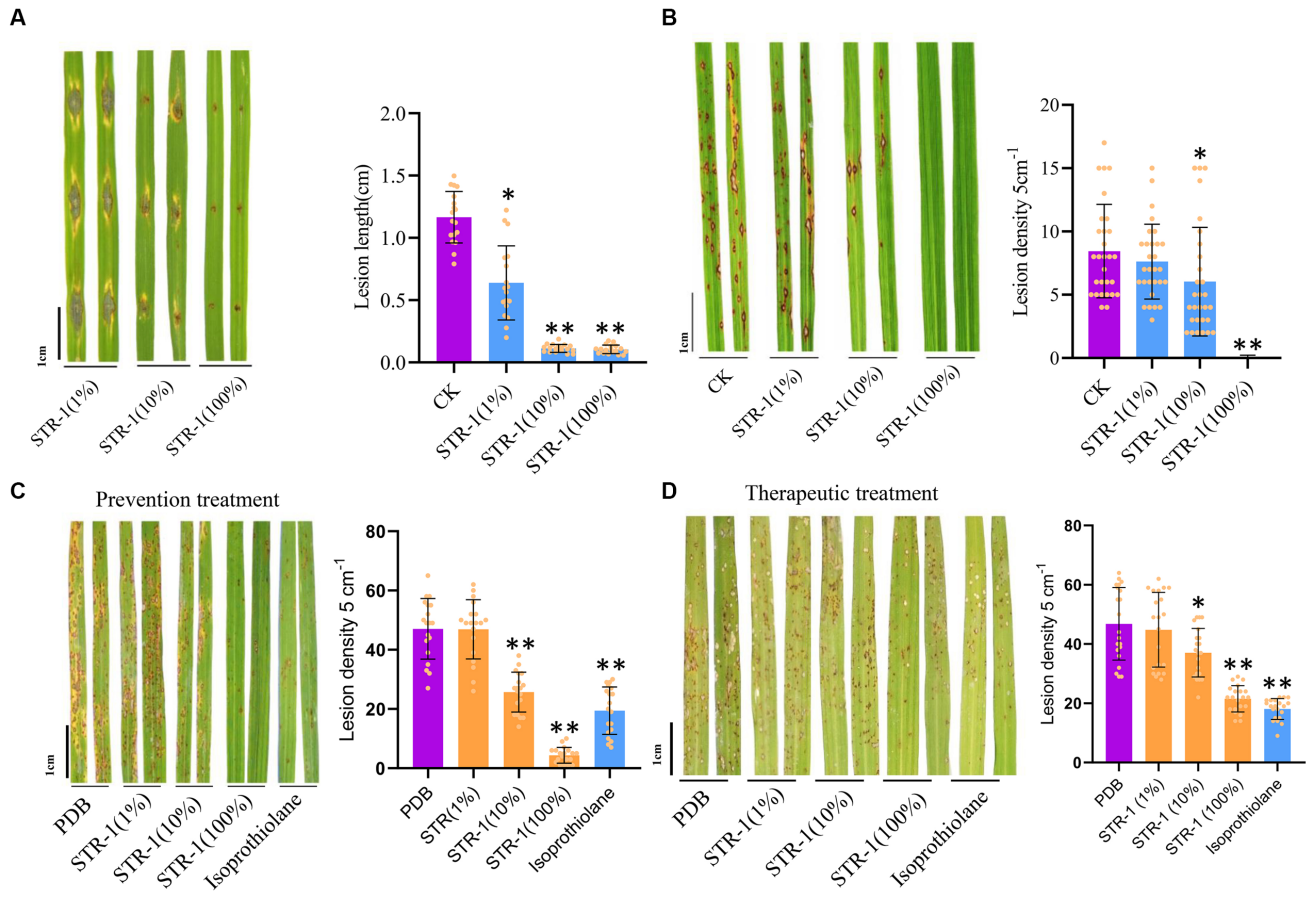
*In vivo* effects of strain STR-1 extract on rice leaves and disease incidence caused by *M. oryzae*. **(A)** Detached leaves infected with Guy11 in spotting inoculation assay with 1, 10, and 100% concentration STR-1 extracts. The PDB was used as a negative control. Length of blast lesion was calculated 5 days after infection (*n* = 15 independent lesions, mean ± s.d., two-sided Student’s *t*-test). **(B)** Rice leaves with concurrent inoculation of 0, 1, 10, and 100% concentration STR-1 extracts and Guy11 spores in a field trial experiment. The PDB was used as a negative control. Blast disease lesion density was quantified from infected leaf segments 5 cm in length 5 d post-infection (*n* = 29 independent leaves; mean ± s.d., two-sided Student’s *t*-test). **(C)** Rice leaves sprayed with 0, 1, 10, and 100% concentration STR-1 extracts, 12 h before inoculation with Guy11 spores in a field trial experiment. The PDB was used as a negative control. Blast disease lesion density was quantified from infected leaf segments 5 cm in length 5 d post-infection (*n* = 29 independent leaves; mean ± s.d., two-sided Student’s *t*-test). **(D)** Rice leaves sprayed with 0, 1, 10, and 100% concentration STR-1 extracts, 12 h after inoculation with Guy11 spores in a field trial experiment. The PDB was used as a negative control. Blast disease lesion density was quantified from infected leaf segments 5 cm in length 5 d post-infection (*n* = 29 independent leaves; mean ± s.d., two-sided Student’s *t*-test). The symbol * and ** stands for *p*  < 0.01 and *p*< 0.05 respectively.

The biocontrol activity of STR-1 against rice blast disease was tested in the greenhouse and field conditions. Under laboratory conditions, leaf punch inoculation assay showed that the average length of blast lesion was 1.16 cm by inoculation with Guy11 conidia suspension in the absence of STR-1 fermentation broth (Figure 3A). However, when exposed to 1% STR-1 fermentation broth, the average length of blast lesion was decreased by approximately 54% to 0.638 cm. Notably, when supplemented into the *M. oryzae* Guy11 conidial suspension, 10 and 100% STR-1 fermentation broth significantly inhibited the occurrence of disease (Figure 3B). We also carried out a field trial to test the effects of STR-1 fermentation broth on controlling blast disease in both prevention and therapeutic treatment, in which STR-1 fermentation broth was, respectively, sprayed before and after Guy11 spore inoculation. In the prevention experiment, 10% STR-1 fermentation broth inhibited the number of blast lesions by over 50% (Figure 3C), displaying the same inhibitory effect as that of isoprothiolane, a fungicide commonly used for controlling rice blast disease in crop production. While in the therapeutic experiment, 100% STR-1 fermentation broth showed the same inhibitory effect as that of isoprothiolane (Figure 3D). These results confirm the effectiveness of the biocontrol bacterium STR-1 in controlling rice blast disease.

### 3.5 Eliciting of rice plant immunity by STR-1

**Figure 4:**
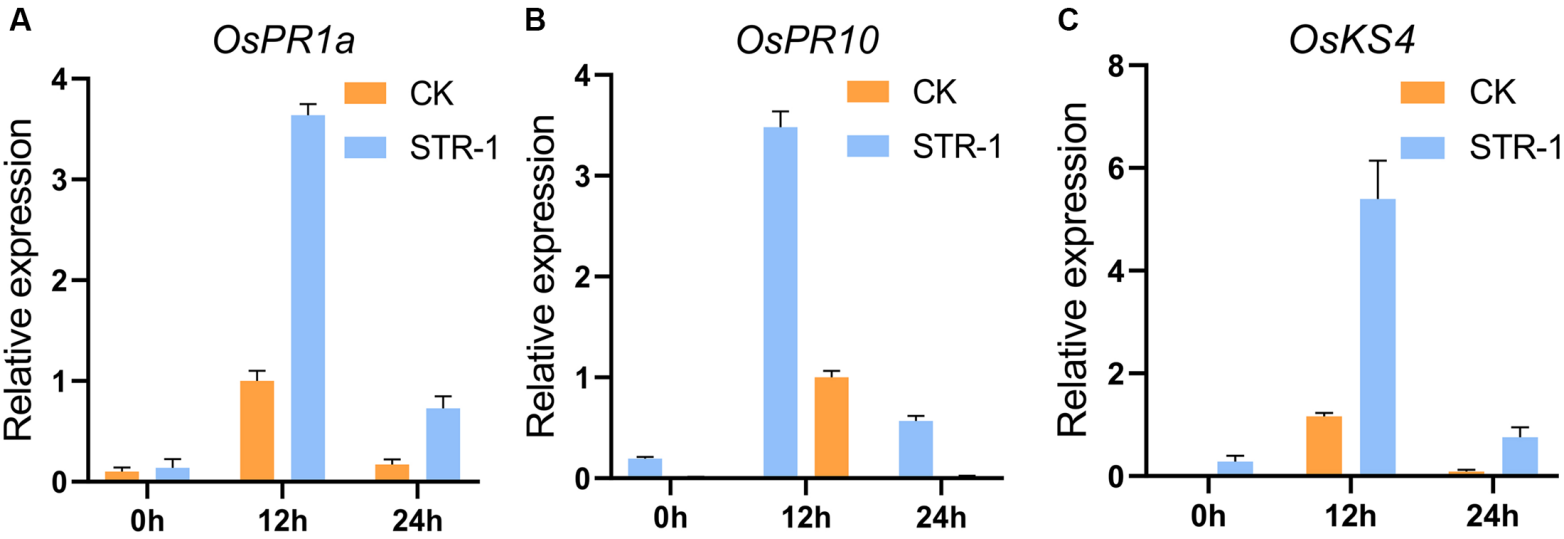
RT-qPCR of PR1a, OsPR10, and OsKS4 in rice leaves sprayed with STR-1 extracts (sterile water as control). **(A)** The salicylic acid inducible *OsPR1a* gene was strongly induced in plants sprayed with STR-1 extracts compared with control. **(B)** The *OsPR10* gene was strongly induced in plants sprayed with STR-1 extracts compared with control. (C) The *OsKS4* gene was strongly induced in plants sprayed with STR-1 extracts compared with control.

In order to detect the expression of genes related to the defense response in rice, we tested the potential effects of STR extracts as plant immunity elicitors by examining the expression of rice defense-related genes. When STR-1 fermentation broths were applied onto the leaves of two-week-old rice, the expression *OsPR1a*, *OsPR10* (pathogenesis-related genes, PRs) and *OsKS4* (synthesis of diterpenoids related genes) was significantly induced after application for 12 h compared to control (Figure 4). The NAC transcript factor 4 was not changed after treatment. STR-1 fermentation broths did not affect rice leaf development, suggesting that the fermentation products of STR-1 were friendly to rice. These results indicate that STR-1 plays a crucial role in rice defense against blast disease likely through eliciting the expression of rice defense-related genes.

### 3.6 Inhibition of STR-1 extract on conidial germination of *Magnaporthe oryzae*

**Figure 5:**
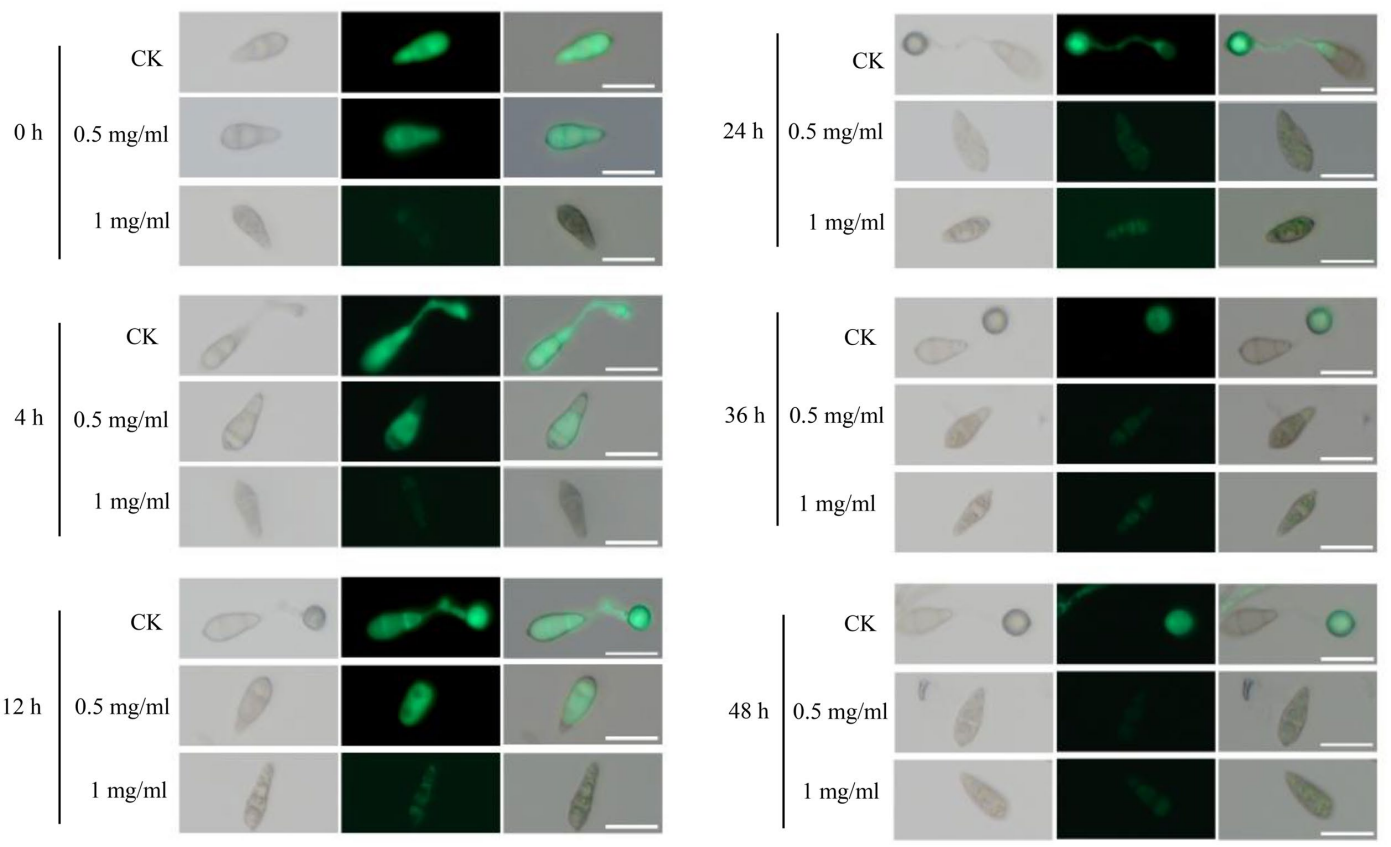
STR-1 extracts have obvious effects on appressorium development of *M. oryzae*. Bright and green fluorescence field micrographs of the appressoria formed by the Guy11 strain expressing GFP on inductive hydrophobic cover slips. After the conidia suspension treated with mock, 0.5 mg/mL and 1 mg/mL concentration STR-1 extracts were inoculated on hydrophobic cover slips and appressoria formation was observed at 0 h, 4 h, 12 h, 24 h, 36 h, and 48 h time intervals. Scale bar = 10 μm.

We then investigated the possible mechanism of the antifungal effects of STR-1 on suppressing rice blast disease. The extracts of STR-1 fermentation broths were added into conidia suspension of a Guy11 strain constitutively expressing green fluorescent protein (GFP) for observing the development of infection-structure development on a hydrophobic surface under microscope. After inoculation for 4 h, conidia started to germinate and form appressoria in the absence of STR-1 extract. However, the conidia supplemented with 0.5 mg/mL STR-1 extract failed to germinate (Figure 5). While in the presence of 1 mg/mL STR-1 extract, the conidia not only failed to germinate, but also were disrupted with the expression of GFP fluorescence. After 12 h, the conidia in the control group formed matured appressoria, but still failed to any infection structure when exposed to 0.5 mg/mL STR-1 extract. The conidia exposed to 1 mg/mL STR-1 extract showed very weak GFP fluorescence, likely due to the inhibition of GFP production by STR-1 extract. These results indicate that STR-1 extract can inhibit spore germination based on the inhibition of protein synthesis.

### 3.7 Bioassay-guided fungicide isolation of STR-1 extract

**Figure 6:**
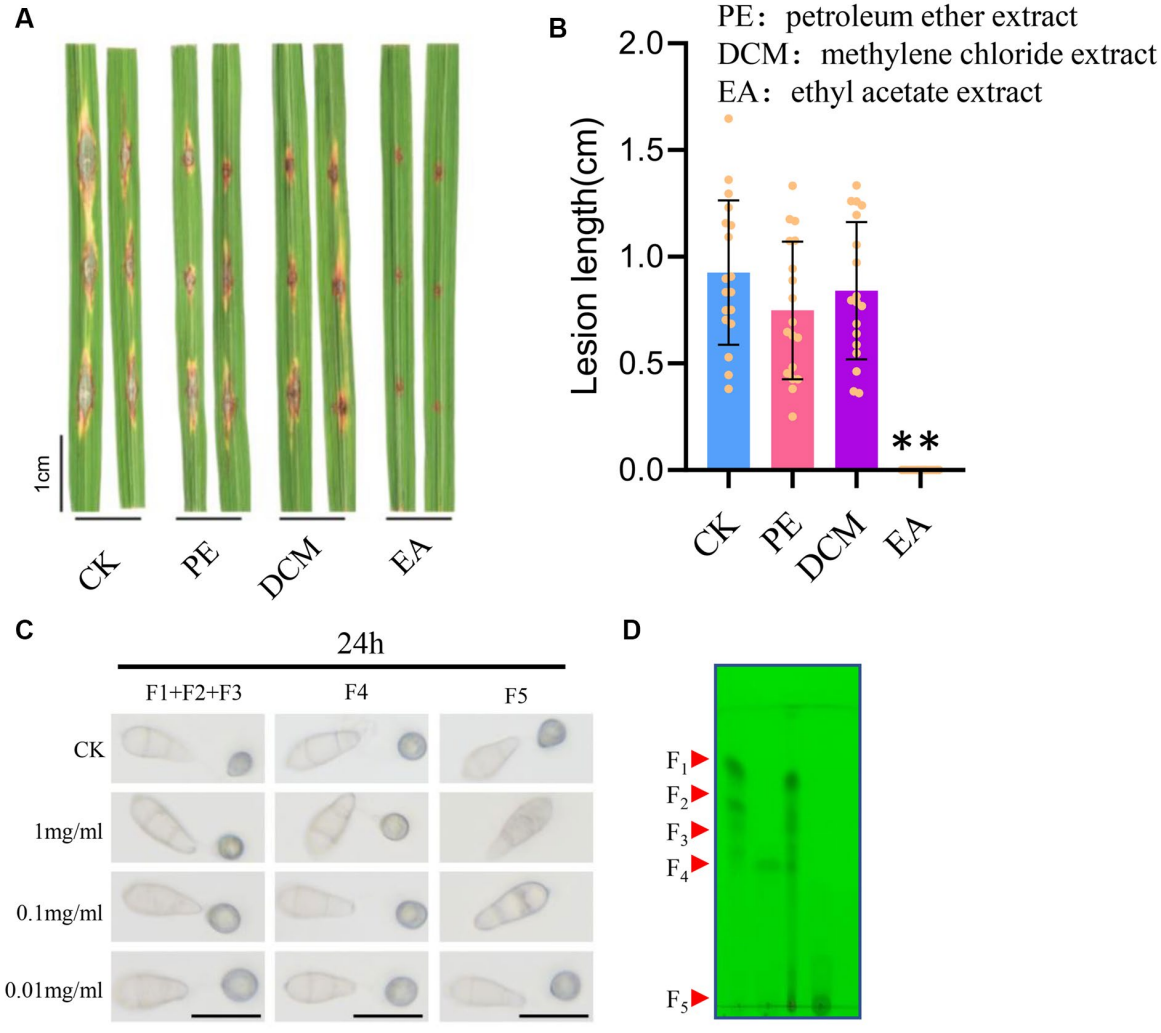
Crude isolation of the active substance of strain STR-1. **(A,B)** Detached leaves infected with Guy11 in spotting inoculation assay with petroleum ether extraction, methylene chloride extract, and ethyl acetate extract of strain STR-1 (After extraction with petroleum ether, dichloromethane, and ethyl acetate, PE, DCM, and EA were concentrated by rotary steamer, respectively). The solvent water was used as a negative control. Length of blast lesion was calculated 5 days after infection (*n* = 15 independent lesions, mean ± S.D., two-sided Student’s *t*-test). **(C)** After, the conidia suspensions treated with F1, F2, F3, F4, or F5 of ethyl acetate extract were inoculated on hydrophobic cover slips and appressoria formation was observed at 24 h. The solvent water was used as a negative control. Scale bar = 10 μm. **(D)** Five segments of ethyl acetate extract of strain STR-1 were separated by column chromatography separation technology, namely F1, F2, F3, F4, and F5. The symbol ** stands for *p*< 0.05.

To further separate the potential antifungal metabolites of STR-1 extracts, we conducted a combination technique with thin layer chromatography and column chromatography. As we have observed, the STR-1 extracts were powerful in inhibiting the conidial germination; we used a bioassay-guided fungicide isolation assay. The STR-1 ethanol extracts were mixed with petroleum ether (PE), methylene chloride (CM), and ethyl acetate (EA), which have different solvent polarities. As shown in Figures 6A,B, the ethyl acetate extracts significantly suppressed blast lesion size on rice leaves when applied into conidial suspension in a punch inoculation assay. Because the biocontrol active metabolites from the strain STR-1 were mostly extracted in ethyl acetate, then the STR-1 ethyl acetate extracts were further separated by column chromatography and thin layer chromatography. As shown in Figures 6C,D, the fraction 5 (F5) was effective at inhibiting spore germination and were further identified by LC–MS, although the ingredients are still complex.

### 3.8 Identification of secondary metabolites from STR-1 extract

**Figure 7:**
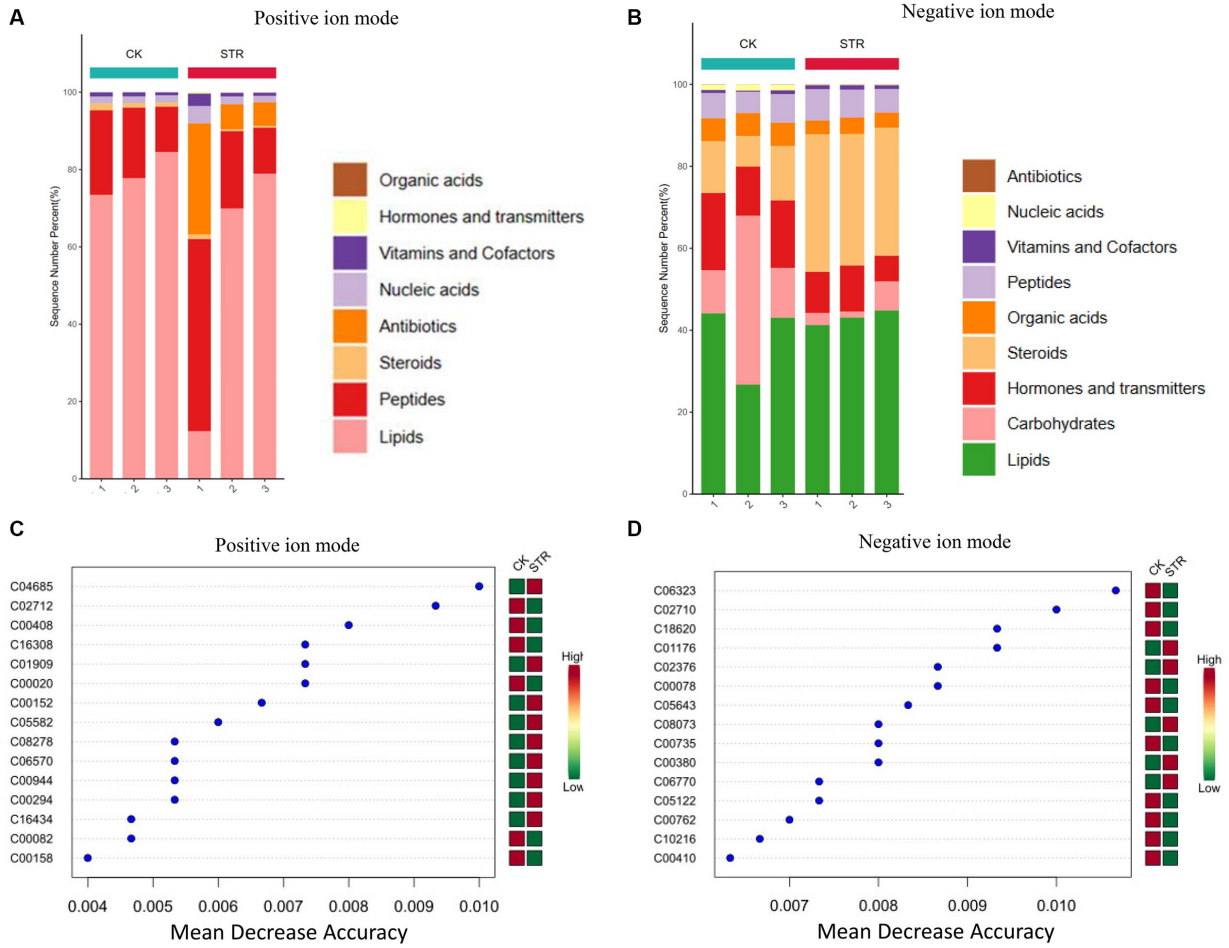
Explore the specific active substance of strain STR-1. Filtration of cultured STR-1 using 1,000 KD dialysis bags was used to detect the difference between the metabolic profiles along with no treatment control. Metabolite extraction of STR-1 and CK was followed by non-targeted metabolomic analysis using liquid chromatography-mass spectrometry (LC–MS) technique. **(A,B)** Cluster analysis of metabolites detected in positive and negative ion modes. **(C,D)** Least squares analysis of metabolites detected in positive and negative ion modes. The symbol ** stands for P<0.05.

To further identify the biocontrol agents of the strain STR-1 extract, we performed a comparative LC–MS/MS before and after fermentation. The nucleic acids, organic acids, hormones and transmitters, and carbohydrates were consumed and produced proteins, steroids, vitamins and cofactors, and antibiotics after fermentation. The antibiotics were produced in great number and detected under the positive and negative ion mode (Figures 7A,B). Principal component analysis (PCA) analysis showed that large amounts of metabolites were produced after dramatic fermentation changes (Figures 7C,D). Characteristic metabolites were further identified as shown in Figures 7C,D by using orthogonal partial least squares discriminant analysis (OPLS-DA) to mine differential metabolites. As shown in Supplementary Table S8, 228 DAMs in the negative ion mode were identified as likely to contribute to the potential antifungal compounds, while 239 DAMs were identified in the positive ion mode. Fold change above 100 was selected and shown in Table 2. The comparative LC–MS proved a valuable clue in determining the secondary metabolites of the strain STR-1 extract. Among these metabolites, 4-oxo-4-[(1-phenylethyl)amino]but-2-enoic acid, 1,3,5-Trimethylpyrazole, and SMA-1 may be the main secondary metabolites which were reported to have antimicrobial activities (Kocyigit-Kaymakcioglu et al., 2008; Zeng et al., 2019).

**Table 2:** Differential secondary metabolite.

Compounds	STR_1	STR_2	STR_3	CK_1	CK_2	CK_3	Fold change	Ion mode
Desthiobiotin	1.77E+09	1.47E+09	3.38E+09	16,178,973	30,109,815	16,519,737	105.5968	−
4-oxo-4-[(1-phenylethyl)amino]but-2-enoic acid	2.59E+08	3.29E+08	3.43E+08	655634.1	1,574,640	797062.8	307.4167	+
1,3,5-Trimethylpyrazole	3.28E+08	4.5E+08	4.47E+08	1,374,674	1,129,636	1,619,425	297.0656	+
SMA-1	5.56E+08	3.84E+08	4.16E+08	2,800,495	1,845,240	1,272,795	229.1286	+
4-Aminobutyric acid	4.54E+08	5.92E+08	2.1E+08	2,537,646	2,864,109	2,579,968	157.3159	+
5,6-Dimethylbenzimidazole	2.49E+08	2.22E+08	2.68E+08	1,835,083	1,638,409	1,568,202	146.5127	+
5-(6-hydroxy-6-methyloctyl)-2,5-dihydrofuran-2-one	4.84E+08	7.69E+08	8.84E+08	6,604,915	5,100,203	6,321,912	118.5265	+
2-Arachidonyl Glycerol ether	17,801,652	94,774,751	60,262,840	570318.8	547078.1	464,742	109.244	+
3-(tert-butyl)-1-methyl-4,5-dihydro-1H-pyrazol-5-one	4.58E+08	5.58E+08	4.24E+08	4,811,175	3,733,950	4,664,899	108.9819	+

## 4 Discussion

The utilization of biocontrol microbial agents is an effective approach for the control and prevention of fungal crop diseases such as rice blast. Although streptomyce is an important source of biocontrol agents from actinomycete, its exploitation and identification of biocontrol metabolites are far from meeting the demands of practical crop production. In this study, a streptomyce strain designated STR-1 was characterized as a new biocontrol agent effective in inhibiting various fungal pathogens, including *M. oryzae*, *R. solani*, *U. virens*, *B. maydis*, and *F. graminearum* (Figure 2). STR-1 can also trigger immune responses in rice (Figure 4), indicating its efficacy in biocontrol in another manner by eliciting rice immunity. Our discovery enriches the biocontrol resources of actinomycete and provides a new avenue for developing biocontrol agents against rice blast and other crop fungal diseases.

Many microbes have been used in biological control. Biocontrol bacteria including *Bacillus subtilisi*, *Pseudomonas* spp., and *Streptomyce* have been widely used in biological control (Park et al., 2006; Ahmed et al., 2022). *Streptomyces* are capable of producing a wide range of secondary metabolites, such as antibiotics. They have thus been considered as an important source of biocontrol agents applied in plant disease control. Despite their value for identifying compounds with outstanding biological control activity, *Streptomyces* produce complex metabolites whose activity are often influenced by various environmental and cultivation conditions. For instance, the efficacy of fermentation broth of *Streptomyce* F2 on inhibiting the growth of anthracnose fungus of peppers was decreased at temperatures above 70°C (Jiang and Song, 2015). In addition, the biocontrol activity of fermentation extract of *Streptomyce* H4 was maintained under ultraviolet light and strong alkaline conditions but was decreased under high temperatures and strong acid conditions (Li et al., 2021a,b). Unlike these previous findings, our study revealed that the fermentation extract of biocontrol strain STR-1 maintained high antifungal activity at temperatures ranging from 40°C to 130°C and exhibited stability within the pH range of 2.0 to 12.0 (Supplementary Figure S1). These results suggest that the antifungal components produced by STR-1 possess excellent stability and likely contribute to the development and utilization of novel biocontrol metabolites.

Many metabolites of microbes can confer induced systemic resistance in plants by activating plant’s immune system for defending biotic or abiotic stresses (Al-wahshi et al., 2023). For example, treatment with biocontrol agents induces the expression of numerous disease-related genes in plants. *Bacillus siamensis* YC-9 increased cucumber resistance against disease caused by *F. oxysporum* f. sp. *cucumerinum* through increasing the activities of defensive enzymes such as peroxidase (POD), polyphenol oxidase (PPO), and phenylalanine aminolase (PAL) in the plant roots (Zhou et al., 2022). When applied to inhibit rice blast disease, *B. siamensis* B-612 fermentation broth can promote peroxidase (POD) activity of rice and the promotion effect peaked 48 h after inoculation (Yang Y. et al., 2023). Our study found that inoculation of STR-1 extract induced the expression of rice disease-related genes such as *OsKS4*, *OsPR10*, and *OsPR1a* (Figure 4), suggesting that certain secondary metabolites produced by STR-1 may play a crucial role in inducing systemic resistance in rice. This finding offers opportunity for the identification and utilization of compounds as plant immune elicitors from STR-1.

*Streptomyces* can synthesize various active secondary metabolites, such as cyclic peptides, alkaloids, terpenes, and polyketides (Zhang and Qian, 2019). The identified antifungal compounds produced by STR-1 likely include 4-oxo-4-[(1-phenylethyl)amino]-2-enonic acid, 1,3,5-trimethylpyrazole, and SMA. SMA belongs to the macrolide class, while 1,3,5-trimethylpyrazole is classified as an alkaloid. These compounds may disrupt crucial biological processes of blast fungus, such as RNA transcription and protein synthesis, such that they can inhibit spore germination, hyphal growth, and other infection-related developmental processes as observed in our study (Figure 5). The identification of these potential biocontrol-effective secondary metabolites provides solid support for the development of new pesticide leading compounds against crop fungal diseases.

## 5 Conclusion

In summary, the identification and characterization of STR-1 as a new strain of *Streptomyces* offers opportunity to discover novel bioactive metabolites for controlling crop fungal diseases. The fermentation extract of STR-1 effectively inhibits the spore germination and hyphal growth of rice blast fungus by suppressing its pathogenicity, with the active secondary metabolites showing high stability in high temperatures and under a strict pH. The ethyl acetate fraction of STR-1 fermentation extract demonstrates outstanding biocontrol activity, likely attributed to its potential active metabolites including 4-oxo-4-[(1-phenylethyl)amino]-2-enonic acid, 1,3,5-trimethylpyrazole, and SMA. The identified biocontrol metabolites of STR-1 and its determined genome sequence are helpful for exploring new compounds with elite biocontrol activity. Further identification and modification of biocontrol-active secondary metabolites, together with functional analysis of the biosynthesis gene cluster in STR-1, will provide an important foundation for conceptual understanding and development of novel strategies for disease control.

## Data availability statement

The genomic sequences of STR-1 have been deposited in National Center for Biotechnology Information under BioProject ID PRJNA1047497. All data are available in the main text or the supplementary materials. All data are additionally available from the authors in other formats as needed upon request.

## Author contributions

WS: Investigation, Writing – original draft, Writing – review & editing. RL: Investigation, Resources, Visualization, Writing – original draft, Writing – review & editing. JW: Data curation, Formal analysis, Investigation, Visualization, Writing – original draft. MY: Formal analysis, Investigation, Resources, Visualization, Writing – original draft. TQ: Funding acquisition, Writing – original draft, Writing – review & editing. GS: Investigation, Resources, Writing – original draft. MH: Resources, Supervision, Writing – original draft, Writing – review & editing. XC: Funding acquisition, Resources, Supervision, Writing – review & editing, Writing – original draft.

## Footnotes

^1^ https://www.megasoftware.net/

^2^ https://antismash.secondarymetabolites.org/

^3^ https://imagej.net/software/imagej2/
