# Supplementary material for: Characterization of a broad-spectrum antifungal strain, Streptomyces graminearus STR-1, against Magnaporthe oryzae
Source: Front Microbiol. 2024 Apr 8;15:1298781. doi: 10.3389/fmicb.2024.1298781 (PMC11033478; doi:10.3389/fmicb.2024.1298781)
Supplement: Supplementary file 1 [file Data_Sheet_1.docx]

**Table S1 Statistics of sequencing data**

| Read_Num | Total_base | N_rate | GC_Content | Q20_rate(%) | Q30_rate(%) |
| --- | --- | --- | --- | --- | --- |
| 10,087,882 | 1,513,182,300 | 0.0007 | 71.68 | 96.78 | 92.58 |

**Table S2 Data statistics of genome assembly**

| Sample | Property | Contig | Scaffold |
| --- | --- | --- | --- |
|  | Total sequence number | 89 | 70 |
|  | Total sequence length | 8,446,604 | 8,446,604 |
|  | Max sequence length | 638,818 | 638,818 |
|  | Min sequence length | 420 | 513 |
|  | N20 | 404,546 | 486,528 |
| STR-1 | N50 | 214,874 | 290,096 |
|  | N90 | 62,567 | 121,131 |
|  | N number | 0 | 910 |
|  | N rate | 0 | 0.00011 |
|  | GC content % | 71.79 | 71.79 |
|  | Sequences greater than 1kb | 77 | 60 |

**Table S3 Data statistics of open reading frame prediction**

| Property | Value |
| --- | --- |
| ORF num | 7399 |
| ORF total length | 7331952 bp |
| ORF density | 0.876 genes per kb |
| Longest ORF length | 23460 bp |
| ORF average length | 990.94 bp |
| Intergenetic region length | 1114652 bp |
| ORF/Genome(coding percentage) | 86.80% |
| Intergenetic length/Genome | 13.20% |
| GC content in ORF region | 72.20% |
| GC content in intergenetic region | 69.09% |

**Table S4 Data statistics for non-coding RNA predictions**

| Sample | Type | Copy Number | Avg. length (bp) | Total length (bp) | percent of genome (%) |
| --- | --- | --- | --- | --- | --- |
|  | 5S rRNA | 2 | 111 | 222 | 0.0026 |
|  | 16S rRNA | 2 | 1,278 | 2,556 | 0.0303 |
| STR-1 | 23S rRNA | 3 | 1,729 | 5,189 | 0.0614 |
|  | tRNA | 68 | 76 | 5,226 | 0.0619 |
|  | ncRNA | 49 | 112 | 5,527 | 0.0654 |

**Table S5 Overview of functional annotation of protein-coding genes**

| Sample | Annotation in Database | No. Of Genes | % |
| --- | --- | --- | --- |
|  | NR | 7,244 | 97.9051 |
|  | eggNOG | 6,294 | 85.0655 |
| STR-1 | KEGG | 2,556 | 34.5452 |
|  | Swiss-Prot | 3,756 | 50.7636 |
|  | GO | 5,061 | 68.4011 |

**Table S6 Statistics of egg NOG (COG) classification annotation results**

| COG categories | | categories function | ORF number | | | % |
| --- | --- | --- | --- | --- | --- | --- |
| A | | RNA processing and modification | 1 | | | 0.0135 |
| B | | Chromatin structure and dynamics | 0 | | | 0 |
| C | | Energy production and conversion | 358 | | | 4.8385 |
| D | | Cell cycle control, cell division, chromosome partitioning | 38 | | | 0.5136 |
| E | | Amino acid transport and metabolism | 423 | | | 5.7170 |
| F | | Nucleotide transport and metabolism | 105 | | | 1.4191 |
| G | | Carbohydrate transport and metabolism | 392 | | | 5.2980 |
| H | | Coenzyme transport and metabolism | 178 | | | 2.4057 |
| I | | Lipid transport and metabolism | 214 | | | 2.8923 |
| J | | Translation, ribosomal structure and biogenesis | 205 | | | 2.7706 |
| K | | Transcription | 632 | | | 8.5417 |
| L | | Replication, recombination and repair | 203 | | | 2.7436 |
| M | | Cell wall/membrane/envelope biogenesis | 224 | | | 3.0274 |
| N | | Cell motility | 1 | | | 0.0135 |
| O | | Posttranslational modification, protein turnover, chaperones | 185 | | | 2.5003 |
| P | | Inorganic ion transport and metabolism | 268 | | | 3.6221 |
| Q | | Secondary metabolites biosynthesis, transport and catabolism | 250 | | | 3.3788 |
| R | | General function prediction only | 0 | | | 0 |
| S | Function unknown | | | 2,138 | 28.8958 | |
| T | Signal transduction mechanisms | | | 325 | 4.3925 | |
| U | Intracellular trafficking, secretion, and vesicular transport | | | 30 | 0.4055 | |
| V | Defense mechanisms | | | 123 | 1.6624 | |
| W | Extracellular structures | | | 1 | 0.0135 | |
| Y | Nuclear structure | | | 0 | 0 | |
| Z | Cytoskeleton | | | 0 | 0 | |
| - | Not in eggNOG | | | 1105 | 14.9345 | |

**Table S7. STR-1 of antifungal secondary metabolites**

| Type | Most similar known cluster | Similarity |
| --- | --- | --- |
| thiopeptide,LAP,terpene | hopene | 92% |
| NRPS,phosphonate,NRPS-like,betalactone | leupeptin Pr/leupeptin Ac | 100% |
| other,NRPS | SMA-1 | 89% |
| ectoine | ectoine | 100% |
| NRP-metallophore,NRPS | mirubactin | 78% |
| terpene | geosmin | 100% |
| NRPS,T1PKS | pentamycin | 100% |
| NI-siderophore | desferrioxamin B/desferrioxamine E | 83% |
| melanin | melanin | 60% |
| T3PKS | flaviolin/1,3,6,8-tetrahydroxynaphthalene | 100% |
| T2PKS,T1PKS | spore pigment,Polyketide | 83% |
