# Supplementary material for: Characterization of a broad-spectrum antifungal strain, Streptomyces graminearus STR-1, against Magnaporthe oryzae
Source: Front Microbiol. 2024 Apr 8;15:1298781. doi: 10.3389/fmicb.2024.1298781 (PMC11033478; doi:10.3389/fmicb.2024.1298781)
Supplement: Supplementary file 3 [file Data_Sheet_3.docx]

>16S_rRNA_7 16S ribosomal RNA (partial)

TAACTACGTGCCAGCAGCCGCGGTAATACGTAGGGCGCAAGCGTTGTCCGGATTTATTGG

GCGTAAAGCGAGCGCAGGCGGTTTTTTAAGTCTGATGTGAAAGCCTTCGGCTCAACCGAA

GAAGTGCATCGGAAACTGGGAAACTTGAGTGCAGAAGAGGACAGTGGAACTCCATGTGTA

GCGGTGAAATGCGTAGAGATCTGGAGGAATACCGGTGGCGAAGGCGGCCCCCTGGACGAA

GACTGACGCTCAGGTGCGAAAGCGTGGGGAGCAAACAGGATTAGATACCCTGGTAGTCCA

CGCTGTAAACGATGTCGACTTGGAGGTTGTGCCCTTGAGGCGTGGCTTCCGGAGCTAACG

CGTTAAGTCGACCGCCTGGGGAGTACGGCCGCAAGGTTAAAACTCAAATGAATTGACGGG

GGCCCGCACAAGCGGTGGAGCATGTGGTTTAATTCGATGCAACGCGAAGAACCTTACCTA

CTCTTGACATCCAGAGAATTTGCTAGAGATAGCTTAGTGCCTTCGGGAACTCTGAGACAG

GTGCTGCATGGCTGTCGTCAGCTCGTGTTGTGAAATGTTGGGTTAAGTCCCGCAACGAGC

GCAACCCTTATCCTTTGTTGCCAGCGATTCGGTCGGGAACTCAAAGGAGACTGCCGGTGA

TAAACCGGAGGAAGGTGGGGATGACGTCAAGTCATCATGGCCCTTACGAGTAGGGCTACA

CACGTGCTACAATGGCGTATACAAAGAGAAGCGACCTCGCGAGAGTAAGCGGACCTCATA

AAGTACGTCGTAGTCCGGATTGGAGTCTGCAACTCGACTCCATGAAGTCGGAATCGCTAG

TAATCGTAGATCAGAATGCTACGGTGAATACGTTCCCGGGCCTTGTACACACCGCCCGTC

ACACCATGGGAGTGGGTTGCAAAAGAAGTAGGTAGCTTAACCTTCGGGAGGGCGCTTACC

ACTTTGTGATTCATGACTGGGGTGAAGTCGTAACAAGGTAACCGTAGGGGAACCTGCGGT

TGGATCACCTCCTT

>MN368134.1 Uncultured Streptomyces sp. clone SH 16S ribosomal RNA gene, partial sequence

AACACATGCAAGTCGAGCGGTAGCACAGGAGAGCTTGCTCTCCGGGTGACGAGCGGCGGACGGGTGAGTAATGTCTGGGAAACTGCCTGATGGAGGGGGATAACTACTGGAAACGGTAGCTAATACCGCATAACGTCTACGGACCAAAGTGGGGGACCTTCGGGCCTCACGCCATCAGATGTGCCCAGATGGGATTAGCTAGTAGGTGGGGTAATGGCTCACCTAGGCGACGATCCCTAGCTGGTCTGAGAGGATGACCAGCCACACTGGAACTGAGACACGGTCCAGACTCCTACGGGAGGCAGCAGTGGGGAATATTGCACAATGGGCGCAAGCCTGATGCAGCCATGCCGCGTGTGTGAAGAAGGCCTTAGGGTTGTAAAGCACTTTCAGCGAGGAGGAAGGGTAGTGTGTTAATAGCACATTGCATTGACGTTACTCGCAGAAGAAGCACCGGCTAACTCCGTGCCAGCAGCCGCGGTAATACGGAGGGTGCAAGCGTTAATCGGAATTACTGGGCGTAAAGCGCACGCAGGCGGTTTGTTAAGTCAGATGTGAAATCCCCGCGCTTAACGTGGGAACTGCATTTGAAACTGGCAAGCTAGAGTCTTGTAGAGGGGGGTAGAATTCCAGGTGTAGCGGTGAAATGCGTAGAGATCTGGAGGAATACCGGTGGCGAAGGCGGCCCCCTGGACAAAGACTGACGCTCAGGTGCGAAAGCGTGGGGAGCAAACAGGATTAGATACCCTGGTAGTCCACGCTGTAAACGATGTCGATTTGGAGGTTGTGCCCTTGAGGCGTGGCTTCCGGAGCTAACGCGTTAAATCGACCGCCTGGGGAGTACGGCCGCAAGGTTAAAACTCAAATGAATTGACGGGGGCCCGCACAAGCGGTGGAGCATGTGGTTTAATTCGATGCAACGCGAAGAACCTTACCTACTCTTGACATCCAGAGAATTCGCTAGAGATAGCTTAGTGCCTTCGGGAACTCTGAGACAGGTGCTGCATGGCTGTCGTCAGCTCGTGTTGTGAAATGTTGGGTTAAGTCCCGCAACGAGCGCAACCCTTATCCTTTGTTGCCAGCGATTCGGTCGGGAACTCAAAGGAGACTGCCGGTGATAAACCGGAGGAAGGTGGGGATGACGTCAAGTCATCATGGCCCTTACGAGTAGGGCTACACACGTGCTACAATGGCGTATAC

AAAGAGAAGCGAACTCGCGAGAGCAAGCGGACCTCATAAAGTACGTCGTAGTCCGGATTGGAGTCTGCAACTCGACTCCATGAAGTCGGAATCGCTAGTAATCGTAGATCAGAATGCTACGGTGAATACGTTCCCGGGCCTTGTACACACCGCCCGTCACACCATGGGAGTGGGTTGCAAAAGAAGTACGTAGCTTAACCTTCGGGAGGGCGCTTACCACTTTGTGATTCATGACTGGGGTGAA
